# Spatial Presentations, but Not Response Formats Influence Spatial-Numerical Associations in Adults

**DOI:** 10.3389/fpsyg.2018.02608

**Published:** 2018-12-18

**Authors:** Ursula Fischer, Stefan Huber, Hans-Christoph Nuerk, Ulrike Cress, Korbinian Moeller

**Affiliations:** ^1^Department of Sport Science, University of Konstanz, Konstanz, Germany; ^2^Thurgau University of Teacher Education, Kreuzlingen, Switzerland; ^3^Leibniz-Institut für Wissensmedien, Tübingen, Germany; ^4^Department of Psychology, University of Tuebingen, Tübingen, Germany

**Keywords:** spatial-numerical associations, numerical processing, magnitude representation, embodied numerosity, SNARC effect

## Abstract

According to theories of embodied numerosity, processing of numerical magnitude is anchored in bodily experiences. In particular, spatial representations of number interact with movement in physical space, but it is still unclear whether the extent of the movement is relevant for this interaction. In this study, we compared spatial-numerical associations over response movements of differing spatial expansion. We expected spatial-numerical effects to increase with the extent of physical response movements. In addition, we hypothesized that these effects should be influenced by whether or not a spatial representation of numbers was presented. Adult participants performed two tasks: a *magnitude classification* (comparing numbers to the fixed standard 5), from which we calculated the Spatial Numerical Association of Response Codes (SNARC) effect; and a *magnitude comparison* task (comparing two numbers against each other), from which we calculated a relative numerical congruity effect (NCE), which describes that when two relatively small numbers are compared, responses to the smaller number are faster than responses to the larger number; and vice versa for large numbers. A SNARC effect was observed across all conditions and was not influenced by response movement extent but increased when a number line was presented. In contrast, an NCE was only observed when no number line was presented. This suggests that the SNARC effect and the NCE reflect two different processes. The SNARC effect seems to represent a highly automated classification of numbers as large or small, which is further emphasized by the presentation of a number line. In contrast, the NCE likely results from participants not only classifying numbers as small or large, but also processing their relative size within the relevant section of their mental number line representation. An additional external presentation of a number line might interfere with this process, resulting in overall slower responses. This study follows up on previous spatial-numerical training studies and has implications for future spatial-numerical trainings. Specifically, similar studies with children showed contrasting results, in that response format but not number line presentation influenced spatial-numerical associations. Accordingly, during development, the relative relevance of physical experiences and presentation format for spatial-numerical associations might change.

## Introduction

Knowledge about numbers and numerical concepts is acquired through interaction with the world around us (e.g., Fischer and Brugger, 2011; Moeller et al., 2012; Myachykov et al., 2014). Although a predisposition to perceive and process magnitudes might be innate or at least present very early in life (e.g., Xu et al., 2005), numerical knowledge is also acquired through physical experiences. Perception of magnitude information, which is often associated with spatial expansion, shapes the way in which magnitudes and numbers are processed (e.g., Fischer and Brugger, 2011; Lindemann and Fischer, 2015). Additionally, physical interaction also seem to play a major role in the acquisition of numerical abilities (e.g., Fischer et al., 2017).

The theoretical account that explains the aforementioned phenomena, also referred to as *embodied numerosity* (Domahs et al., 2010) has received increasing research interest in recent years. Especially finger counting has been described as an example of bodily experiences associated with processing of numerical information and was even argued to lead to a specific finger-based representation of numerical magnitude that persists into adulthood (Fischer and Brugger, 2011; Roesch and Moeller, 2015).

Importantly, there is evidence suggesting that associations between numbers and space can be influenced not only by bodily experiences with fingers but also the whole body (Fischer et al., 2016). In the current study, we not only investigated the effects of bodily movement on the processing of numbers, but also the interplay of movement and visual perception. In the following, we first introduce measures of spatial-numerical associations before giving an overview of the literature on embodied numerosity and embodied trainings. We then summarize previous findings on the interplay of presentation and response in spatial-numerical associations before describing the current study.

### Spatial-Numerical Associations

Numerical magnitude has long been thought to be associated with physical space. This association can either be between numerical and physical extensions (e.g., Henik and Tzelgov, 1982; Siegler and Opfer, 2003; Moeller et al., 2009) or between numbers and a particular direction in space (e.g., Dehaene et al., 1993; see Cipora et al., 2015; Patro et al., 2015a, for this distinction). Regarding spatial directionality, number magnitudes are assumed to be spatially represented along a *mental number line* (see Göbel et al., 2011; Fischer and Shaki, 2014, for reviews). This systematic association of numbers and space seems to develop early in life (e.g., Patro and Haman, 2012; Macchi Cassia et al., 2016; McCrink et al., 2017), and become more and more consolidated until adulthood (Kaufmann et al., 2008; de Hevia and Spelke, 2009).

The mental number line is assumed to be activated automatically whenever number magnitude information is processed (Tzelgov et al., 1992; Dehaene et al., 1993; Rubinsten and Henik, 2005). However, this activation was observed to depend on how relevant number magnitude is for a specific task, and also on how magnitudes are presented and responded to (Nuerk et al., 2005; van Dijck et al., 2009; Fischer et al., 2016). Certain behavioral effects have been established as indicators of spatial-numerical associations, two of which we considered in the present study.

#### The SNARC Effect

One of the most well-known indicators for spatial-numerical associations is the SNARC effect (Spatial Numerical Association of Response Codes, Dehaene et al., 1993). It describes the finding that in Western cultures, small numbers are associated with the left side of space, whereas large numbers are associated with the right side of space (see Wood et al., 2008, for a meta-analysis). Accordingly, when Western participants are asked to respond to smaller numbers with the left hand and to large numbers with the right hand (congruent response direction), they are faster and less error prone than when the response direction is reversed so they have to respond to smaller numbers with the right hand and to larger numbers with the left hand (incongruent response direction, Dehaene et al., 1993). For example, when comparing numbers from 1 to 9 to a fixed standard of 5 in a *magnitude classification task*, responses to the number ‘2’ are made faster with the left than with the right hand, whereas responses to the number ‘8’ are made faster with the right than with the left hand. In the original interpretation, Dehaene et al. (1993) argued that this pattern of results stemmed from an automatic activation of the left-to-right oriented mental number line, with left/right hand responses being either congruent or incongruent with the position of small/large numbers on the mental number line. Alternative accounts, however (e.g., van Dijck and Fias, 2011; Gevers et al., 2010) argue that the SNARC effect does not result from mental number line activation, but rather from working memory processes, or from a verbal coding of the numbers. For example, numbers could be verbally coded as semantically SMALL or LARGE, and the semantic codes could then be associated with the left and right side of space. This verbal coding would be sufficient to illicit a SNARC effect, without the necessity for an explicit processing of the number magnitude (e.g., Gevers et al., 2006c; Proctor and Cho, 2006; Santens and Gevers, 2008; Imbo et al., 2012; see also Schroeder et al., 2017 for a discussion of linguistic influences on the SNARC effect).

#### The Numerical Congruity Effect

Another indicator of spatial-numerical associations is the relative numerical congruity effect (NCE) described by Fischer et al. (2016) and based on the congruity effect described by Dehaene (1989). In contrast to the SNARC effect, this effect does not result from changing response assignments for ‘smaller’ and ‘larger’ responses. To measure this effect, participants again compare the magnitude of two numbers (e.g., comparing 2–4). They are instructed to respond with the left hand when the target number is smaller than the other number, and with the right hand when it is larger than the other number. However, both of the numbers can vary in size, necessitating an actual *magnitude comparison* between the two numbers rather than a simple classification as smaller or larger than 5. Here, the effect is also calculated by comparing congruent and incongruent responses. However, congruity is not determined by a change in the response direction as for the SNARC effect. Rather, congruity results from a match or mismatch between the absolute size of the number that is responded to (i.e., small or large) and its relative size to the comparison standard (i.e., smaller or larger). For example, in a congruent comparison, participants have to decide whether the number ‘2’ is smaller or larger than the number ‘4.’ The correct response is ‘smaller,’ and is made with the left hand, congruently with the position of ‘2’ on the left side of the number line. However, when switching the numbers and comparing ‘4’ to the standard ‘2,’ the *relative* size of the number ‘4’ compared to the number 2 is *larger*, and therefore, a response has to be made with the right hand. However, in the range from 0 to 9, the *absolute* size of ‘4’ is *small*. The resulting incongruence between the absolute and relative magnitude leads to slower and more error-prone responses. The effect can be explained by assuming that for a number to be classified as small within the range 1–9, the mental number line representation of the continuum 1–9 may be co-activated in addition to the magnitude of the to-be-compared numbers. A similar explanation was proposed previously for related effects (i.e., the *semantic congruity effect*, see e.g., Banks et al., 1976; Cantlon and Brannon, 2005). It is therefore possible that the NCE, due to its reliance on activating the entire relevant number range on the mental number line, presents a more direct measure of spatial-numerical associations than the SNARC effect.

### Embodied Numerosity and Embodied Trainings

Recently, spatial-numerical associations have received increasing research interest following numerous studies showing that they are associated with bodily movements (Moeller et al., 2012; Patro et al., 2015b). Indeed, as elaborated on in theories of embodied numerosity (Domahs et al., 2010) bodily movements play an important role in arithmetic and numerical processing, most notably through the use of fingers for counting and representing numbers (e.g., Fischer and Brugger, 2011; Fabbri and Guarini, 2016; Suggate et al., 2017). Most children use their fingers during early numerical development, and the way in which numbers are represented on one’s fingers has a substantial impact on the development of spatial-numerical associations (Wasner et al., 2014).

Recent research suggests, however, that bodily movements that interact with spatial-numerical associations generalize from the hands to the whole body (e.g., Fischer, 2003; Schwarz and Müller, 2006; Hartmann et al., 2012; Klein et al., 2014; Shaki and Fischer, 2014). For example, Shaki and Fischer (2014) observed that when participants were asked to make lateral turns to the left or right while walking and generating random numbers, they were more likely to turn left after generating a small number, and to turn right after generating a large number. This finding can be explained by participants associating small numbers with full-body movements to the left and large numbers with full-body movements to the right.

Following the previous studies investigating interactions between numbers and the body, full-body movements have been used to not only measure spatial-numerical associations (Fischer et al., 2016), but also to boost the training success of spatial-numerical trainings in so-called embodied training approaches (e.g., Dackermann et al., 2017). In most conventional spatial-numerical trainings, participants are trained in a numerical task that also incorporates spatial aspects. For example, children are trained to count numbers which are ordered from left to right or to estimate the position of numbers on a presented number line (e.g., Siegler and Ramani, 2009; Kucian et al., 2011; Sella et al., 2016). The goal of these trainings is to help children understand numerical concepts or to improve their mathematical skills. Trainings that highlight the spatial ordering of numbers are often more beneficial than trainings that do not, as children show more pronounced improvement in the trained tasks but also in untrained transfer tasks (e.g., Siegler and Ramani, 2009). Embodied spatial-numerical training approaches take this concept one step further, as they combine this spatial-numerical task presentation with a spatial full-body response movement. Accordingly, children are trained to respond to a spatial-numerical task with a full-body movement. For example, Fischer et al. (2011) presented kindergartners with a number located on a number line and then asked them to decide whether a second number was smaller or larger. This training was more effective when children responded with their entire body (by jumping to the left for smaller and to the right for larger decisions) than when they responded manually. Children specifically improved more in number line estimation (i.e., they were able to more accurately locate numbers on an empty number line) as well as their understanding of counting principles (i.e., they were better able to count backward or in steps of two). Training concepts such as these were already implemented with different types of training tasks (such as number line estimation) and with different age groups (ranging from kindergarten to second grade). In all previous embodied spatial-numerical trainings studies, a task-relevant full-body movement in accordance with the direction of the mental number line further increased training effects (for overviews see Fischer et al., 2015a; Dackermann et al., 2017).

However, the specific working mechanisms of embodied spatial-numerical trainings are not yet fully understood. Previously, Fischer et al. (2011) argued that in accordance with theories of perception-action integration (e.g., Hommel et al., 2001; Hommel, 2009), the combined spatial features of the full-body response movement and the presentation of a number line increased the activation of the mental number line. This increased activation was then assumed to lead to a deeper processing of the task, in turn increasing training gains (for further training studies see also Link et al., 2013; Fischer et al., 2015b; Dackermann et al., 2016).

While the success of full-body spatial-numerical trainings has been investigated and supported several times, the respective training studies also raised the question of whether it was indeed the combination of full-body movements and spatial presentation of training content that increased training effects, or whether either the spatially distributed presentation along a number line or the full-body response would have sufficed. The underlying working mechanisms of these trainings were first investigated in an experimental study by Fischer et al. (2016), in which different response and presentation formats were compared to measure their influence on the strength of spatial-numerical associations. As the current study builds upon this previous study, we now describe it in more detail.

### The Interplay of Presentation and Response in Spatial-Numerical Associations

Fischer et al. (2016) first investigated the differential effects of bodily responses on spatial-numerical associations in elementary school children. They expected that a full-body response movement that corresponds to the direction of the mental number line would elicit stronger spatial-numerical associations than a verbal response format. Furthermore, they controlled for the effect of an additional explicit presentation of a number line. In doing so, they evaluated spatial-numerical associations by the two effects described above – the SNARC effect and the NCE. They hypothesized that these effects should be modulated systematically by response and presentation formats. More specifically, they expected the most pronounced effects when full-body responses and the explicit presentation of a number line were combined.

They found that, at least in elementary school children, the strength of spatial-numerical associations was only influenced by response format, but not by the presentation of a number line. In particular, a SNARC effect was observed irrespective of response conditions, whereas the NCE was only observed in conditions requiring physical response movements. Thereby, physical response movements seemingly increased spatial-numerical associations, but only when magnitude processing was necessary as reflected by the NCE in magnitude comparison with a variable standard.

However, while Fischer et al. (2016) differentiated between responses conducted with foot movements and verbal responses, it remained unclear whether maybe a manual response movement as used in typical SNARC experiments would have been sufficient to elicit a NCE. Furthermore, the bodily and verbal responses in the previous study differed in another relevant aspect. While the bodily responses were made horizontally (i.e., to the left and right) to correspond to the horizontal orientation of the presented number line, the verbal responses were made vertically so as not to correspond to the horizontal number line orientation. That is, participants responded by saying ‘up’ and ‘down’ rather than ‘left’ and ‘right.’ This confound between spatial orientation and the modality of the response might have limited the generalizability of the results. Because of these two caveats of the previous study, only limited conclusions could be drawn about whether full-body movement influences spatial-numerical associations. Accordingly, more fine-grained research is necessary to determine whether the degree of bodily movement can influence spatial-numerical associations. Furthermore, spatial-numerical associations keep developing after elementary school age (Ninaus et al., 2017). It is therefore plausible to assume that influences of response and presentation format as investigated by Fischer et al. (2016) may look differently in adults, when spatial-numerical associations are stable and do not need further development. Accordingly, the current study was designed to address these previous issues.

### The Current Study

Measuring both SNARC effect and NCE and building directly on the study by Fischer et al. (2016), we examined the strength of spatial-numerical associations for different types of presentation and response formats in adults. As previously observed by Fischer et al. (2016) in children, we expected that deeper magnitude processing should lead to more pronounced SNARC effects and NCEs.

The extent of bodily movement was varied in three different response formats: Verbal, manual, and full-body responses. Although responses were all spatially oriented (to the left or right), we expected that active bodily movement should increase spatial-numerical associations, whereas they should be smaller in verbal responses as previously observed (Fischer et al., 2016). In accordance with the effects of passive full-body movements on numerical processing (Hartmann et al., 2012), we further expected that spatial-numerical associations should be more pronounced for full-body compared to manual responses, because these full-body responses provide additional vestibular information that is absent in manual responses.

Also in line with previous work, we varied stimulus presentation. In embodied numerical training studies, activation of the mental number line was often additionally enhanced by presenting a number line along with the task (for overviews see Fischer et al., 2015a, 2017). However, a previous experimental study with elementary school children found no differences in spatial-numerical effects whether a number line was presented or not (Fischer et al., 2016). Accordingly, the question whether number line presentation thus leads to more pronounced spatial-numerical associations in addition to the response format has not yet been fully resolved. Therefore, the adult participants in our study also received two different presentation formats. The to-be-compared numbers were either presented along a horizontal number line ranging from 0 to 10 or above each other without a number line.

Finally, we were interested in whether response format and mode of stimulus presentation would interact in affecting spatial-numerical associations. Assuming that both presentation and response format impact spatial-numerical associations, there should be an additive effect on SNARC effect and NCE, with the strongest effects being present when a number line was presented and a full-body movement is required as the response.

Previous results also indicated that the SNARC effect might not only reflect spatial-numerical associations but also aspects of verbal coding. In turn, the SNARC effect should occur regardless of response format. However, the NCE might be more exclusively determined by spatial-numerical associations, which is why we expected it to increase steadily with the extent of the required response movement.

## Materials and Methods

### Participants

Prior to testing, we conducted an *a priori* power analysis to determine the necessary number of participants using the program G^∗^Power 3.1.9.2 (Faul et al., 2009). We assumed small effect sizes of around f = .1 for both the SNARC effect and NCE, and wanted to acquire a statistical power of 0.90. Accordingly, we entered 2 × 3 × 2 = 12 measurements for our within-subject design and assumed a strong correlation between our repeated measures of 0.8. The power analysis suggested a sample size of at least 37 participants.

Forty-five university students took part in the study. Out of these, five had to be excluded from the analysis due to missing data. In two cases, the voice key software did not recognize the participants’ voice onset correctly, and in three cases, technical difficulties lead to missing data files. Out of the remaining 40 participants (13 male; age: *M* = 21.6 years, *SD* = 2.9 years, range = 18–30 years), 35 reported being right-handed. Written informed consent was obtained from participants and the study was approved by the local ethics committee.

### Tasks and Effects

**FIGURE 1:**
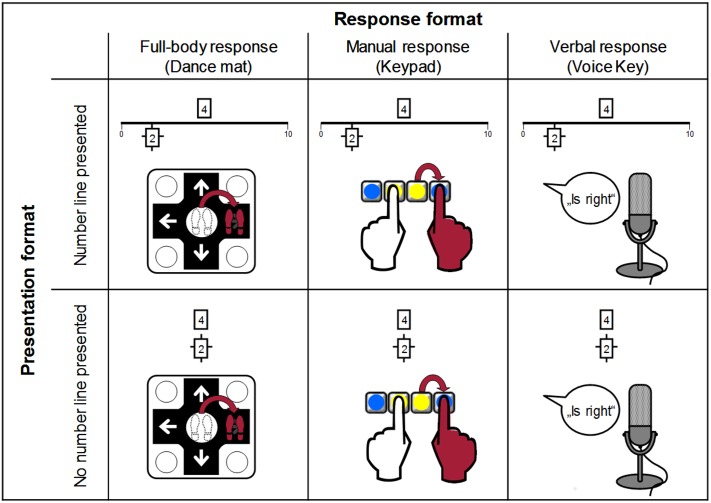
Variation of response and visual presentation formats demonstrated on the magnitude classification of 4 vs. 2. Here, the number 2 is marked as the standard for comparison, so participants had to decide whether 4 is larger or smaller than 2.

To measure SNARC effect and NCE, we used two types of numerical comparison tasks. In both tasks, participants decided whether the magnitude of a target number was smaller or larger than a simultaneously presented comparison standard. To distinguish the target from the standard, the rectangle surrounding the standard was marked by additional cross-shaped lines (see Figure 1).

The paradigms differed with respect to the comparison standard, which was fixed in the SNARC task (*magnitude classification*) and variable in the NCE task (*magnitude comparison*). This difference in comparison standards impacts the relevance of magnitude processing: While in magnitude classification, a number only has to be classified as small or large, magnitude comparison requires an actual magnitude comparison between the two numbers (Dehaene, 1989).

#### Magnitude Classification Task (Fixed Standard)

In magnitude classification, numbers had to be compared to the fixed comparison standard 5 (see also Nuerk et al., 2005; Gevers et al., 2006c, 2010). To evaluate the SNARC effect, we varied response direction congruity (congruent vs. incongruent). In the number line congruent direction, participants responded to the left for ‘smaller’ decisions and to the right for ‘larger’ decisions, whereas in the number line incongruent direction^1^, they responded to the right for ‘smaller’ decisions and to the left for ‘larger’ decisions. The SNARC effect was then calculated by comparing the incongruent and congruent response direction condition (for a similar procedure see Mapelli et al., 2003; Gevers et al., 2006a,b).

In the magnitude classification task, 5 was used as the fixed standard and 1, 4, 6, 9 as targets. All numbers were presented at an equal frequency (each number 12 times per condition) and in random order.

#### Magnitude Comparison Task (Variable Standard)

In magnitude comparison, the comparison standard was varied like the comparison probe from trial to trial (see also Banks et al., 1976; Cantlon and Brannon, 2005). Other than in magnitude classification, participants always responded ‘smaller’ to the left and ‘larger’ to the right. To evaluate the NCE, we varied whether the correct response (‘smaller’ or ‘larger’) corresponded to the absolute magnitude (small or large) of the to-be-compared number and thus, its position on the mental number line. For example, compared to the standard 4, the number 2 requires a ‘smaller’ response to the left. Because within the relevant range of 1–9, 2 is a small number that is located on the left side of the mental number line, this leftward ‘smaller’ response is congruent with the mental number line position of 2. In contrast, when 4 is compared to the standard 2, this would call for a ‘larger’ response to the right. Now this response is incongruent with the position of the small number 4 on the left side of the mental number line. Implementing these two types of trials, the NCE was analyzed by comparing incongruent and congruent trials.

The standard was a flexible number in the range between 1 and 9 (excluding 5), and both numbers of a pair were always either smaller than 5 or larger than 5. We used all possible number pairs in the range from 1 to 9, which resulted in a total of 12 number pairs (smaller than 5: 1–2, 1–3, 1–4, 2–3, 2–4, 3–4; larger than 5: 6–7, 6–8, 6–9, 7–8, 7–9, 8–9). Each pair was presented eight times per condition, four of which as a congruent pairing (e.g., 2–4) and four as an incongruent pairing (e.g., 4–2), again in random order.

### Procedure and Apparatus

Participants were tested individually in a university lab. Each participant came in for two sessions that lasted approximately 55 min each. In each session, participants were given the opportunity to take a break in between the different response conditions. To keep experimental conditions as comparable as possible across response conditions, all tasks were presented by projecting them onto a wall in front of participants at a distance of 2.5 m. Tasks were programmed in Java Eclipse and ran on a standard notebook (Fujitsu Siemens Lifebook T 4010).

The three different response formats were implemented using three different types of response media. In the *verbal response condition*, participants responded by speaking their answer into a microphone that was placed on a desktop and adjusted in height for each participant. Participants responded by either saying ‘Is left.’ (Translated from the German ‘Ist links.’) or ‘Is right.’ (German: ‘Ist rechts.’). A voice key programmed into the experimental software registered response latencies by detecting the onset of speech, while response accuracy was recorded manually by the experimenter. The verb ‘is’ was added to allow for the voice key software to capture the actual speech onset analogously for both responses, i.e., without phonemic differences influencing the measured voice onset times.

In the manual response condition, participants were seated at a table and responded on an external numeric keypad with the index fingers of both hands. To avoid the numbers on the keypad interfering with the response and to help participants remember the correct response keys, circular stickers were placed on the keys to cover the numbers. Each trial started with the participant’s fingers on two adjacent keys of the keypad, located centrally in the second row from the bottom (keys ‘2’ and ‘3’) and marked with yellow stickers. To respond, participants had to press the key to the left (key ‘1’) or right (‘enter’ key) from the starting point of the respective index finger, which were marked with blue stickers (see also Figure 1).

In the full-body response condition, we used a digital dance mat (Positive Gaming Impact Dance Pad^2^) with fields arranged in a 3 × 3 layout. Participants responded by hopping from the central field to the right or left field of the dance mat depending on their decision. Both the external keypad and the dance mat were connected to the notebook via USB.

Visual presentation (number line or no number line) was varied by either presenting the comparison standard correctly placed on a number line (endpoints marked 0 and 10) with the to-be-compared number placed centrally above the number line or by presenting both numbers above each other without a number line (see Figure 1).

### Design

The experimental manipulations resulted in a 2 × 3 × 2 design for both tasks. For magnitude classification (fixed standard), the factors were response direction (SNARC compatible/incompatible), response format, and presentation format. For magnitude comparison (variable standard), the factors were congruity, response format, and presentation format. Half of the participants started with magnitude classification (fixed standard), while the other half started with magnitude comparison (variable standard). The order of permutations of the factors was balanced between participants. To this end, we generated 2 × 3 × 2 different task sequences and randomly assigned participants to one of them.

In each of the two tasks, participants completed 576 trials. These trials were presented in 12 blocks of 48 trials in magnitude classification (2 response directions, 3 response formats, and 2 presentation formats), and in 6 blocks of 96 trials in magnitude comparison (3 response formats and 2 presentation formats). Note that response direction was varied in blocks in the magnitude classification task. However, congruity in the comparison task was not blocked and varied on a trial by trial basis, as it was determined by the relationship between the presented magnitudes and thus not dependent on a change of response direction.

### Analysis

Prior to analyses, any response times (RT) below or above 3 standard deviations of each participant’s individual mean and all RT faster than 200 ms were removed to control for outliers. Only RT for correct responses were analyzed. RT for magnitude classification (fixed standard) and magnitude comparison (variable standard) were then entered into a within-subject repeated measures design. We conducted separate 2 × 3 × 2 (2 *response direction*/*numerical congruity* × 3 *response formats* × 2 *presentation formats*) repeated-measures analyses of variance for magnitude classification (testing the SNARC effect with a fixed standard) and magnitude comparison (testing the NCE with a variable standard). The presence of a significant SNARC effect/NCE was determined by a main effect of response direction/congruity. Any significant effects involving the three-staged factor response format were followed up by pairwise comparisons between the three response formats to determine the origins of the interaction. Analyses were conducted using SPSS 25 (IBM Corp, 2017).

### Data Availability

Datasets are available on request.

## Results

Overall, participants were faster in the magnitude classification task with a fixed standard (*M* = 851 ms, *SD* = 272 ms) than in the magnitude comparison task with a variable standard (*M* = 1052 ms, *SD* = 301 ms). Because error rates were very low in both tasks (magnitude classification: 4.2%, magnitude comparison: 5.4%), error rates were not analyzed any further.^3^

**FIGURE 2:**
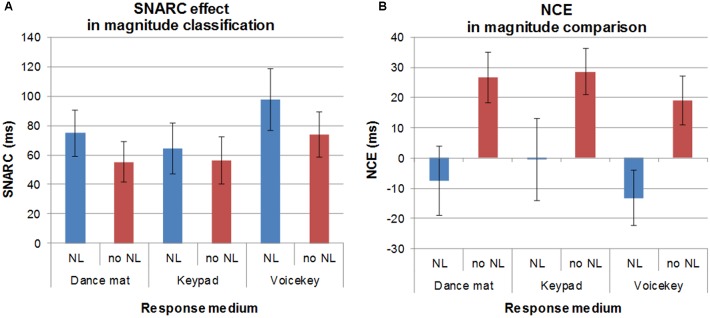
Mean effects of spatial-numerical associations in each condition in the magnitude classification task (fixed standard) measuring the SNARC effect **(A)** and magnitude comparison task (variable standard) measuring the NCE **(B)**. Error bars represent ± 1 SEM.

Figure 2 gives an overview of the mean effects (SNARC effect/NCE) in RT in each condition of both tasks. An overview over raw RT in each condition can be found in the Supplementary Material.

### Results Magnitude Classification (Fixed Standard): SNARC Effect

Analyses revealed a significant overall SNARC effect as indicated by a main effect of response direction *F*(1,39) = 36.47, *p* = 0.000, $\eta_{p}^{2}$ = 0.48. Participants were faster in the SNARC compatible direction (849 ms) than in the SNARC incompatible direction (915 ms). There was also a significant main effect of response format, *F*(2,78) = 388.66, *p* = 0.000, $\eta_{p}^{2}$ = 9.09; RT_fullbody_ = 1165 ms vs. RT_manual_ = 708 ms vs. RT_verbal_ = 773 ms. The full-body movement condition led to slower responses than both the manual and the verbal condition, and responses in the manual condition were faster than in the verbal condition.^4^

Number line presentation also yielded a main effect of RT, as responses were slower when a number line was presented than when it was not, *F*(1,39) = 5.61, *p* = 0.023, $\eta_{p}^{2}$ = 0.13; RT_nl_ _presented_ = 889 ms vs. RT_nonl_ = 875 ms.

Only the interaction between response direction and presentation format was significant, *F*(1,39) = 4.69, *p* = 0.037, $\eta_{p}^{2}$ = 0.11. The SNARC effect was more pronounced when a number line was presented than when no number line was presented.

No other interactions reached significance (all *F* < 2.59, all *p* > 0.082).

Because we had hypothesized that the SNARC effect should differ between the response formats, but found no significant interaction between response direction and response format to support this hypothesis, we followed up the ANOVA with a Bayesian analysis. This analysis tested the alternative hypothesis that there should be an interaction against the null hypothesis of no interaction between response direction and response format. Using the SPSS_BAYES_ANOVA expansion pack for SPSS Statistics 25.0 (IBM Corp, 2017), we calculated the Bayes factor (alternative/null) for the interaction, which suggested that the data were 0.047:1 in favor of the null hypothesis, or 21.3 times more likely to occur under a model without the interaction than a model including the interaction. According to previously suggested interpretation criteria for the Bayes factor (e.g., Wetzels et al., 2011), this presents strong evidence in favor of the null hypothesis.

### Results Magnitude Comparison (Variable Standard): NCE

In magnitude comparison, we observed no overall significant NCE as indicated by a non-significant main effect of congruity, *F*(1,39) = 2.38, *p* = 0.131, $\eta_{p}^{2}$ = 0.06; RT_congruent_ = 1087 ms vs. RT_incongruent_ = 1095 ms. Because we had expected a main effect of congruity, we followed this up with a Bayesian analysis. This analysis (alternative/null) revealed that the data were 0.38:1 in favor of the null hypothesis, or 2.62 times more likely under the null than under the alternative hypothesis. This presents only anecdotal evidence in favor of the null hypothesis that there is no overall NCE in the data (e.g., Wetzels et al., 2011), and therefore, the null effect should be interpreted with caution.

As in the magnitude classification task with a fixed standard, there was a main effect of response format, *F*(2,78) = 221.72, *p* = 0.000, $\eta_{p}^{2}$ = 0.85; RT_fullbody_ = 1369 ms vs. RT_manual_ = 935 ms vs. RT_verbal_ = 969 ms. Responses on the dance mat were slower than manual and verbal responses, whereas verbal and manual response condition did not differ in response speed. Presentation format did not yield a main effect, *F*(1,39) = 1.13, *p* = 0.294, $\eta_{p}^{2}$ = 0.03.

However, congruity interacted significantly with presentation format, *F*(1,39) = 10.58, *p* = 0.002, $\eta_{p}^{2}$ = 0.21. *Post hoc* comparisons of congruent and incongruent RT indicated that when a number line was presented, there was no significant NCE, *t*(39) = 0.95, *p* = 0.348, but there was a significant regular NCE when no number line was presented, *t*(39) = 4.28, *p* = 0.000, with incongruent responses (834 ms) being slower than congruent ones (803 ms).

Furthermore, the interaction between response format and presentation format was significant, *F*(2,78) = 3.68, *p* = 0.030, $\eta_{p}^{2}$ = 0.09. Following this interaction up with *post hoc* pairwise comparisons, we first calculated the differences between the two presentation formats (without vs. with a number line) and compared these across the response formats. There was a significant difference between the full-body and verbal conditions, *t*(39) = 2.55, *p* = 0.015, with full-body comparisons being faster with than without a number line (No number line – number line = 18.76 ms), while verbal responses were faster without than with a number line (No number line – number line = -29.07 ms). Furthermore, there was a marginally significant difference between the full-body and manual conditions, *t*(39) = 1.97, *p* = 0.056, which again can be explained by the full-body responses showing faster responses with than without a number line (No number line – number line = 18.76 ms) compared to the manual condition, where responses were faster without than with a number line (No number line – number line = -23.42 ms). No significant difference was observed between the verbal and manual condition, *t*(39) = 0.33, *p* = 0.747.

No other interactions reached significance (all *F* < 1.4, all *p* > 0.243). Like the SNARC effect, the NCE did not differ significantly between the response formats as indicated by the non-significant interaction between congruity and response format. Again, we therefore followed up the ANOVA with a Bayesian analysis (alternative/null). The analysis revealed that the data were 0.044:1 in favor of the null hypothesis, which corresponds to the data being 22.7 times more likely under a model without the interaction than under a model including the interaction – again indicating strong evidence for the null hypothesis that there is no interaction in the data (e.g., Wetzels et al., 2011).

## Discussion

For the first time, the current study investigated the interplay of response and presentation formats for spatial-numerical associations in adult participants. Following up on previous developmental studies (Fischer et al., 2016), we expected spatial-numerical associations (SNARC effect and NCE) to increase with the extent of left-right physical movements in the response format. Furthermore, we expected that the explicit presentation of a number line should lead to more pronounced spatial-numerical associations. The most pronounced effects were therefore expected for full-body responses in combination with the explicit presentation of a number line. However, our data suggest that these mechanisms may be different in adults compared to children, and that spatial-numerical associations change during development.

Most notably, there were no differences in the strengths of SNARC effect and NCE in the three response conditions. However, unlike in children, adult participants were influenced by the presentation of a number line along with the task, which was not always beneficial. We discuss the theoretical impact of these findings in the following.

### Theoretical Implications

In line with previous work (Fischer et al., 2016), we observed differences in the result patterns for SNARC effect and NCE. In particular, the SNARC effect was again observed in every condition of the magnitude classification task, whereas the NCE was only observed in certain conditions of the magnitude comparison task. However, the SNARC effect differed depending on the presentation format, with number line presentation yielding larger SNARC effects than a presentation without a number line. This influence of number line presentation was not observed in children (Fischer et al., 2016), but seems to indicate an involvement of an underlying mental number line in the occurrence of the SNARC effect in adults.

Regarding the NCE, the picture was more inconsistent, as it was not observed overall, but only when no number line was presented. However, overall RT did not differ depending on number line presentation. A closer inspection of the marginal means revealed that participants performed at roughly the same speed whenever a trial was incongruent (with NL: 1093 ms; without NL: 1098 ms); and even on congruent trials when a number line was presented (1101 ms). However, when a congruent trial was presented without a number line, response speed increased (1073 ms). Accordingly, the absence of a number line seemed to help participants to solve congruent trials faster. This finding might either indicate that participants did not refer to any spatial-numerical directional representation for solving the congruent trials, and therefore benefited from not having to process redundant visual information. Alternatively, participants might in general rely more on their internal mental number line for the magnitude comparison task, potentially ‘zooming in’ on the relevant section of the number line (i.e., 0–5 when comparing 2 and 4), and can do so more efficiently when they do not have to inhibit an externally presented number line of a non-fitting larger range (i.e., 0–10). However, in the latter case, this should also result in processing advantages for incongruent trials with no number line presentation, which was not supported by the data. Here, future studies would be desirable to further differentiate spatial and numerical aspects of the presentation format.

Another unexpected finding was the interaction between presentation and response format in the magnitude comparison task measuring the NCE. Here, we observed that when participants responded with their entire body, number line presentation led to faster responses compared to a presentation without a number line. However, the opposite was observed for verbal and manual responses, which were descriptively slower with than without number line presentation. While unexpected, this result fits in with previous explanations for why embodied numerical trainings for children have been efficient in the past. For example, Fischer et al. (2015b) as well as Link et al. (2013) observed that combining a presentation of a number line with a full-body response increased the effects of number line estimation trainings compared to trainings that included only number line presentation or a full-body response. A possible explanation for these previous results is that when being presented with a number line and responding with the entire body, this creates an embodied experience of moving along the number line. This fit between the presentation and movement was previously argued to improve training effects and could also account for faster reaction times only in this particular condition in our study.

### Practical Implications for Education and Trainings

Previous studies implementing embodied spatial-numerical trainings suggested that combining spatial-numerical presentation (e.g., a number line) with full-body spatial responses could increase training success (for overviews see Dackermann et al., 2017; Fischer et al., 2017). The first study investigating the underlying working mechanisms of these trainings (Fischer et al., 2016) partially confirmed this interpretation, as in fourth-graders, response format was more relevant than the presentation of a number line in influencing spatial-numerical associations. However, the current study showed that for adults, the presentation of a number line seemed to play a more prominent role than the response format. Surprisingly, it seemed to hinder rather than to help performance in most conditions.

Within the context of spatial-numerical trainings, the differences in the findings for children and adults might mean that the relevance of each training component (response and presentation) may vary depending on the age of the participants. This possible effect of age should be taken into account when designing future trainings, as older participants might not benefit from an embodied spatial-numerical training in the same way that the young children in previous studies did. To this point, studies on embodied numerical trainings and their underlying mechanisms have only been conducted with children from kindergarten up to fourth grade. It is possible that for children above this age, a full-body response format might not improve training gains, and a presentation of a number line could even hinder training progress. Considering our results, embodied numerical trainings might not even be effective at all for adult participants. However, seeing as the idea behind embodied spatial-numerical trainings is mostly to convey basic numerical competencies, these trainings are not targeted at adult participants. Future studies will be necessary to determine the age at which a full-body response might no longer be adequate. In this vein, longitudinal studies testing the effects of different types of spatial-numerical trainings throughout childhood development would also be informative.

### Limitations and Future Directions

The current study builds on a previous experimental study conducted by Fischer et al. (2016). However, the different age groups investigated mean that the studies are only partially comparable. Because results vary considerably, future studies are needed to close the age gap. In particular, the comparison between manual and full-body responses has not been investigated in children, for whom response format may play a larger role than for adults as indicated by the results of Fischer et al. (2016).

Another aspect to be considered is that task difficulty was possibly not comparable for children and adults. Although the study by Fischer et al. (2016) tested fourth-graders, who should be very familiar with the number range of 0–10, it is reasonable to assume that responses were even more automated for adult participants, and that spatial-numerical effects differed for this reason as well.

A promising avenue for future research could be to test participants across different age groups, while also combining an experimental approach such as the one implemented in the current study with different types of spatial-numerical trainings. Firstly, comparing different age groups within the same paradigm would be informative with regard to what type of training would be most beneficial at what age. Secondly, by measuring spatial-numerical associations before and after trainings, the relevance of the SNARC effect and NCE as measures of spatial-numerical associations may be further clarified. Furthermore, in case these trainings were to vary in whether they include only a spatial response, a spatial presentation, or both, the effect of each training component on spatial-numerical associations could be distinguished more clearly.

## Conclusion

The present findings indicated that adult participants, unlike children, show stable spatial-numerical associations that are independent of the effector with which a task was performed. This suggests that in adults, the strength of spatial-numerical associations is no longer as strongly associated with bodily experiences. Accordingly, while full-body numerical trainings are beneficial for young children, it is possible that trainings for older participants need to take a different approach.

Contrary to previous results of studies with children, visual presentation seemed to play more of a role in adults. However, it was mostly interfering, suggesting that adults’ magnitude representations are either (1) more abstract (see e.g., Cipora et al., 2016), such that visuo-spatial perceptual support actually introduces additional interfering information, or (2) more flexible (see e.g., Thompson and Siegler, 2010), such that a fixed number line does not help, but actually hinders flexible zooming in on the number line, as previously shown for other types of spatial-numerical information (Huber et al., 2014).

## Ethics Statement

This study was carried out in accordance with the recommendations of the ‘Ethische Richtlinien der Deutschen Gesellschaft für Psychologie e.V.’ (Ethical guidelines of the German Psychological Society) with written informed consent from all subjects. All subjects gave written informed consent in accordance with the Declaration of Helsinki. The protocol was approved by the ‘Lokale Ethikkommission am IWM’ (Local ethics committee at KMRC) in Tuebingen, Germany.

## Author Contributions

UF, SH, H-CN, UC, and KM conceptualized the study and designed the experiment. SH programmed the experiment. UF conducted the study, analyzed the data, and wrote the first draft of the manuscript. UF, H-CN, UC, and KM wrote the manuscript.

## Conflict of Interest Statement

The authors declare that the research was conducted in the absence of any commercial or financial relationships that could be construed as a potential conflict of interest.

## Footnotes

1 Note that we tested German participants, who grow up with a left-to-right reading/writing direction and therefore in their majority associate larger numbers with the right side and small numbers with left. Congruity might be defined differently in participants from right-to-left reading/writing cultures.

2 http://www.positivegaming.com

3 Comparisons between left- and right-handed participants revealed no differences depending on handedness in SNARC effect [*t*(43) = 1.1, *p* = 0.274] or NCE [*t*(43) = -1.4, *p* = 0.159], and therefore left- (*N* = 5) and right-handers (*N* = 40) were analyzed together.

4 Because previous studies (see Wood et al., 2008, for an overview) have suggested that the SNARC effect increases with longer response latencies, an additional analysis controlling for this difference in RT between the response formats was conducted and included in the Supplementary Table 1 for the interested reader.
