# Supplementary material for: Spatial Presentations, but Not Response Formats Influence Spatial-Numerical Associations in Adults
Source: Front Psychol. 2018 Dec 18;9:2608. doi: 10.3389/fpsyg.2018.02608 (PMC6305423; doi:10.3389/fpsyg.2018.02608)
Supplement: Supplementary file 1 [file Table_1.pdf]

Supplementary Material

Spatial Presentations, but not Response Formats Influence Spatial-Numerical Associations in Adults

Ursula Fischer*, Stefan Huber, Hans-Christoph Nuerk, Ulrike Cress, Korbinian Moeller

*** Correspondence:** Ursula Fischer: ursula.fischer@uni-konstanz.de

1 Supplementary Figures

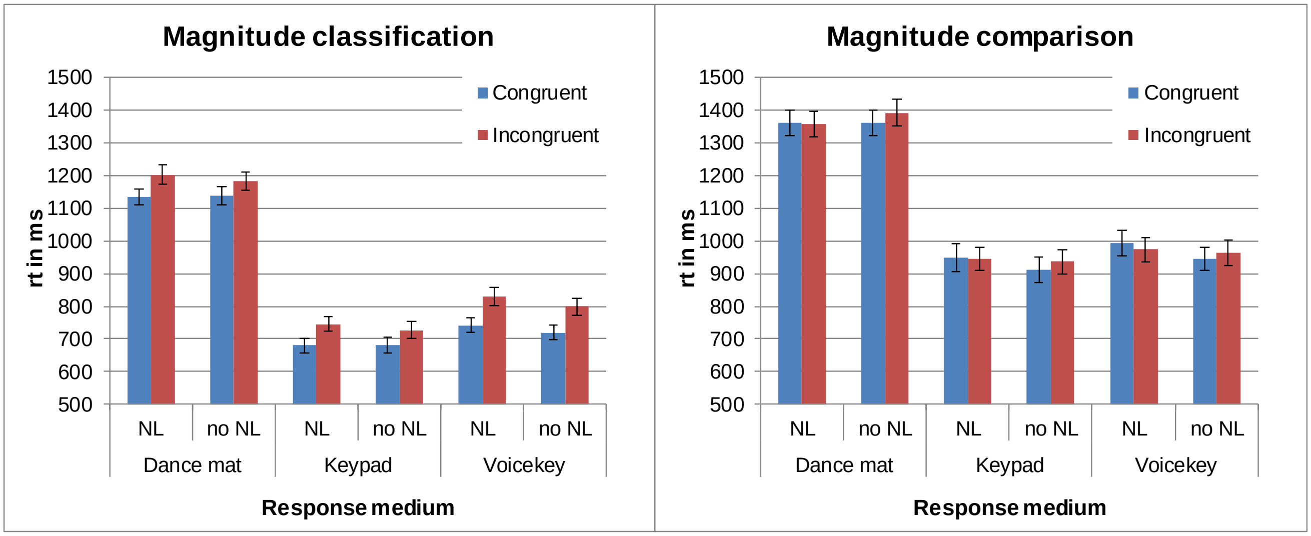

Supplementary Figure 1. Raw RT in each condition of (panel a) the magnitude classification task (fixed standard) and (panel b) the magnitude comparison task (variable standard). Error bars represent ± 1 SEM.

2 Supplementary Tables

Supplementary Table 2. Additional standardized analyses controlling for differences in RT between response formats

	Reported results			Standardized results		
	F	*p*	η_p^2	*F*	*p*	η_p^2
Magnitude comparison (NCE)						
response format	**221.724**	**.000**	**.850**			
congruity	2.375	.131	.057	3.304	.077	.078
presentation format	1.133	.294	.028	1.933	.172	.047
response format * congruity	1.441	.243	.036	2.189	.119	.053

response format * presentation format	**3.684**	**.030**	**.086**	**3.662**	**.030**	**.086**
congruity * presentation format	**10.579**	**.002**	**.213**	**14.482**	**.000**	**.271**
response format * congruity * presentation format	.093	.912	.002	.353	.704	.009
Magnitude classification (SNARC effect)						
response format	**388.666**	**.000**	**.909**			
response direction	**36.471**	**.000**	**.483**	**37.323**	**.000**	**.489**
presentation format	**5.609**	**.023**	**.126**	**7.000**	**.012**	**.152**
response format * response direction	1.576	.213	.039	**3.540**	**.034**	**.083**
response format * presentation format	2.588	.082	.062	**3.541**	**.034**	**.083**
response direction * presentation format	**4.687**	**.037**	**.107**	3.756	.060	.088
response format * response direction * presentation format	.219	.804	.006	.347	.708	.009
	T	*p (two-sided)*		*T*	*p (two-sided)*	
Pairwise comparison of SNARC effects in response formats						
Dance mat vs. Keypad	.087	.931		-1.705	.096	
Dance mat vs. Voice Key	-1.579	.122		**-2.892**	**.006**	
Keypad vs. Voice Key	-1.400	.169		-1.003	.322	

Previous studies (see Wood et al., 2008, for an overview) have suggested that the SNARC effect increases with longer response latencies. We controlled for this by dividing each participant's mean RT per condition (e.g., RT for magnitude comparison in the condition dance mat + congruent + no number line) by their overall mean RT in the respective response format (e.g., RT magnitude comparison for dance mat responses). Thereby, we standardized all means so that response medium no longer had an effect and the average for each response medium was 0. We then calculated both ANOVAs on these standardized data as presented in the manuscript but excluding the main effect of response format (as it was set to 0 by the standardisation). These results were not included in the main manuscript because the analysis eliminates the main effect of the response medium and also might result in an overcorrection of the data.

The results in magnitude comparison (NCE) did not change substantially due to this standardisation. However, the reanalysis changed the result pattern for the magnitude classification data (SNARC effect), which was followed up by pairwise comparisons between the SNARC effects in the three response formats.

In the standardized analysis, we observe an interaction between response format and response direction (i.e., an effect of response format on the SNARC effect). However, pairwise comparisons of the SNARC effect between the response formats suggest that this effect now goes against our initial expectations: The SNARC effect in the dance mat condition is now significantly smaller than in the

voicekey condition, t = -2,89, p = .006, although no other pairwise comparisons reach significance. It is worth noting that the SNARC effect remains significant in every response format.
